# Association of serum high-density lipoprotein cholesterol with microalbuminuria in type 2 diabetes patients

**DOI:** 10.1186/s12944-018-0878-2

**Published:** 2018-10-05

**Authors:** Xun Sun, Ye Xiao, Pei-mei Li, Xiu-yun Ma, Xiao-jie Sun, Wen-shan Lv, Yi-li Wu, Peng Liu, Yan-gang Wang

**Affiliations:** 1 Department of Endocrinology, Weihai Hospital Affiliated to Medical College of Qingdao University, No 51 Guangming road, Huancui District, Weihai, 264200 China; 2 Department of Endocrinology and Metabolic Disease, The Affiliated Hospital of Qingdao University, Qingdao, China; 3 Department of Epidemiology and Health Statistics, Qingdao University, Qingdao, China; 4 Department of Endocrinology, Laiwu Hospital Affiliated to Taishan Medical College, Laiwu, China

**Keywords:** Type 2 diabetes mellitus, High-density lipoprotein cholesterol, Microalbuminuria

## Abstract

**Background:**

The association of serum high-density lipoprotein cholesterol (HDL-C) with microalbuminuria in type 2 diabetes mellitus (T2DM) remains controversial. Therefore, a cross-sectional study was conducted on patients with T2DM to investigate the relationship of HDL-C with microalbuminuria.

**Methods:**

A total of 524 participants with T2DM were recruited in this cross-sectional study. The patients were divided into four groups according to serum HDL-C quartile. A nonparametric test was employed to assess the relationships across quartiles with clinical parameters and demographics. Multivariate logistic regression analysis was further performed.

**Results:**

Of the 524 patients, 138 (26.3%) were found to have microalbuminuria by urinary albumin excretion rate determination. Serum HDL-C levels in microalbuminuria group were significantly lower than those in non-microalbuminuria group (1.04 (0.90–1.21) vs. 1.10 (0.94–1.31) mmol/L, *P* = 0.002). The nonparametric test for trend showed that the prevalence of microalbuminuria was significantly reduced for subjects of the fourth quartile of HDL-C compared to the first to third quartile (13.5% vs. 33.1%, 28.6%, 29.4%, *P* = 0.001). Multivariate logistic regression showed that subjects within the highest quartile of HDL-C had lower odds of microalbuminuria than those within the lowest quartile of HDL-C (OR = 0.17, 95% CI 0.15–0.52, *P* = 0.004).

**Conclusions:**

Higher levels of serum HDL-C were associated with decreased rates of microalbuminuria in T2DM patients.

**Electronic supplementary material:**

The online version of this article (10.1186/s12944-018-0878-2) contains supplementary material, which is available to authorized users.

## Background

With the increasing incidence of diabetes, especially type 2 diabetes mellitus (T2DM), the number of patients with severe complications such as diabetic nephropathy is also on the rise [1]. The International Diabetes Federation Diabetes Atlas estimated that a total of 425 million had diabetes in 2017, and this number is expected to rise to 629 million by 2045 [2]. Diabetic nephropathy (DN) is a major cause of chronic kidney disease which may eventually lead to end-stage renal disease (ESRD). The early prevention, diagnosis and treatment are very important for DN, considering the lack of effective treatment modalities [3]. At present, microalbuminuria, an indicator of glomerular and renal tubular damage as well as endothelial cell dysfunction, is considered to be an early predictor of DN and is associated with a high cardiovascular disease risk [4]. It is widely accepted that microalbuminuria precedes the development of macroalbuminuria and progressive deterioration of renal function to ESRD [5]. In 2006, the DEMAND study showed that the global prevalence of microalbuminuria in patients with T2DM was 39%, and the highest prevalence of albuminuria was found for Asian and Hispanic patients [6]. In our country, a Chinese representative survey conducted by Jia W et al. reported a microalbuminuria prevalence of 22.8% [7]. The annual transition rate was 2–5% from normal urinary albumin excretion to microalbuminuria [8]. Therefore, identifying risk factors for the development of microalbuminuria may provide a basis for the design of effective interventions, not only for DN, but also for other cardiovascular events.

Previous studies have identified hyperglycemia and hypertension as important risk factors for DN [9]. However, it is unlikely that these two factors are the only determinants of DN. For example, the risk for DN among patients with T2DM remains high, despite proper management of blood glucose levels and blood pressure [10]. This residual risk in patients with T2DM indicates that genetic, metabolic, and environmental factors, such as ethnicity, obesity, lipid abnormalities, insulin resistance, pancreatic β-cell function, smoking etc., may also be involved in the pathogenesis of DN. Patients with T2DM typically have dyslipidemia that is characterized by low high-density lipoprotein cholesterol (HDL-C) levels [11]. HDL-C has a reverse transport of cholesterol, anti-inflammatory and antioxidant effects. It has been shown that lower levels of HDL-C may promote the development of microalbuminuria and DN. But the sample size was small and the races among the subjects were different, thus leading to inconsistent results [12–14]. To address discrepancies in the literature, we carried out this study to assess the association of serum HDL-C levels with microalbuminuria in T2DM patients.

## Methods

### Subjects

A total of 659 T2DM patients were included in the study, all of which were selected from our hospital between January 2013 and January 2014. The diagnosis of diabetes followed the 2012 American Diabetes Association diagnostic criteria [15]. Microalbuminuria was defined as urinary albumin excretion rate (UAER) of 20–200 μg/min for at least two out of three 24-h-urine samples. Diabetic retinopathy (DR) was diagnosed from fundus photography according to the EURODIAB recommendations [16]. The patients with pregnancy (*n* = 8), macroalbuminuria (*n* = 43), non-diabetic kidney disease (*n* = 28), urinary-tract infection (*n* = 21), autoimmune disease (*n* = 12), severe heart failure (*n* = 3), liver disease (*n* = 4), malignancies (*n* = 4), and acute complications of diabetes mellitus (*n* = 12) were excluded, and 524 qualified patients were finally enrolled. Of the 524 patients, there were 272 males and 252 females, who were aged 30–75 years. They had a diabetes duration of 0.5–40 years. All patients underwent an interview and provided a history of drinking, smoking and medication. The protocol was designed in adherence to the Declaration of Helsinki and was approved by the ethics committee of the Qingdao University affiliated hospital (No. 2012017). All patients signed the informed consent.

### Anthropometric and laboratory data

Waist circumference (WC) was determined at the midpoint of the lowest rib and the iliac crest. Blood pressure of the patients was measured for a consecutive 3 times with an interval of 5 min. Reported values were the means of those three measurements. The patients were fasted overnight for 8-10 h, and venous blood specimens were harvested for determination of lipid concentrations, creatinine (Cr), fasting plasma glucose (FPG), fasting C peptide, and glycated hemoglobin (HbA1c). The three 24-h-urine samples were collected on different days to determine UAER. Serum HDL-C and low-density lipoprotein cholesterol (LDL-C) (immune turbidimetric methods), serum triglycerides (TG) and total cholesterol (TC) (enzymatic methods), serum creatinine (Cr) (Jaffe methods), fasting plasma glucose (FPG) concentrations (the glucose oxidase method), and UAER (coomassie brilliant blue methods) were measured on a Hitachi 7600 automatic biochemical analyzer (Japan). C-peptide was assessed by electrochemiluminescence assay (Roche E601 automatic immunity analyzer, Switzerland). Glycated hemoglobin (HbA1c) was measured by ion exchange high performance liquid chromatography (Bio-Rad D10 automatic glycosylated hemoglobin, USA). HOMA-B was evaluated according to the formula below: 20 × fasting C-peptide (pmol/L) / (FPG (mmol/L) -3.5). HOMA-IR was evaluated based on the formula below: fasting C-peptide (pmol/L) × FPG (mmol/l) / 22.5 [17]. Two-field stereoscopic fundus photographs (45°) of each patient were taken using free mydriatic fundus cameras (Canon CR-DGi, Japan), and were evaluated by a single trained ophthalmologist.

### Statistical analysis

Stata (Version 11.0) software was employed for statistical analyses. Continuous variables were expressed as median with interquartile ranges, and categorical data were expressed by percentages. The Mann-Whitney U test was used to compare the continuous variables between the microalbuminuria group and non-microalbuminuria group. The chi-square test was employed to compare the categorical data between the two groups above. In addition, the patients were further divided into four subgroups according to quartiles of HDL-C. The associations across quartiles of HDL-C with baseline characteristics and demographics were estimated using a non-parametric test. Multivariate logistic regression was performed. Statistical significance was defined as *P* <  0.05 (two-tailed).

## Results

### Clinical feature comparision between the microalbuminuria group and non-microalbuminuria group

Among those 524 patients with T2DM, 138 (26.3%) had microalbuminuria, as confirmed by UAER examination. Patients with microalbuminuria had significantly higher systolic blood pressure (SBP), diastolic blood pressure (DBP), TG, Cr, BMI, WC, HbA1c, FPG, HOMA-IR, drinking rates, ACE inhibitor and calcium channel blockers administration rates, UAER, and the ratio of DR, but lower HOMA-B compared with those with non-microalbuminuria (All *P* <  0.05). The serum HDL-C levels of subjects in the microalbuminuria group were significantly lower than those in the non-microalbuminuria group [1.04 (0.90–1.21) vs. 1.10 (0.94–1.31) mmol/L, *P* = 0.002] (Table 1**)**.

**Table 1:** Comparison of clinical characteristics between the Microalbuminuria group and non-Microalbuminuria group

Characteristics	Microalbuminuria group	non-Microalbuminuria group	^a^*P* value
n	138	386	
Female (%)	53.3	46.1	0.066
Age (years)	63 (55–73)	62 (55–70)	0.468
SBP (mmHg)	140 (130–153)	132 (120–150)	0.002
DBP (mmHg)	84 (80–90)	80 (75–90)	0.024
TC (mmol/L)	4.7 (3.9–5.3)	4.7 (4.0–5.5)	0.356
TG (mmol/L)	1.5 (1.0–2.3)	1.3 (0.9–1.9)	0.016
HDL-C (mmol/L)	1.04 (0.90–1.21)	1.10 (0.94–1.31)	0.002
LDL-C (mmol/L)	2.6 (2.1–3.1)	2.7 (2.1–3.3)	0.401
Cr (umol/L)	89 (77–103)	84 (74–94)	0.001
BMI (Kg/m^2^)	26.4 (24.6–28.7)	25.8 (23.7–28.4)	0.058
WC (cm)	99 (92–105)	95 (90–102)	0.008
Duration of diabetes (years)	10 (6–15)	10 (5–15)	0.091
HbA1c (%)	9.0 (7.9–10.4)	8.5 (7.3–10.3)	0.040
FPG (mmol/L)	8.1 (6.9–10.1)	7.4 (6.2–9.0)	< 0.001
Fasting C peptide (ng/ml)	1.9 (1.3–2.5)	1.7 (1.1–2.5)	0.316
HOMA-IR	248 (136–354)	194 (114–316)	0.019
HOMA-B	2547 (1755–3958)	3059 (1975–4658)	0.007
Smoking (%)	28.3	22.5	0.143
Drinking (%)	16.1	11.9	0.025
Diabetic retinopathy (%)	64.4	21.7	< 0.001
Diabetes medications (%)			
Metformin	60.1	56.5	0.414
Sulfonylureas	19.6	24.8	0.213
Thiazolidinediones	9.4	15.1	0.105
Acarbose	66.7	69.2	0.584
Exenatide	2.2	1.0	0.323
Saxagliptin	5.1	3.9	0.556
Insulin	81.2	77.0	0.259
Antihypertensive medications (%)			
ACE inhibitor	52.9	42.0	0.028
β-Blocker	21.0	17.5	0.360
Calcium channel blockers	33.3	23.5	0.024
Lipid-lowing medications (%)			
Statins	50.0	47.5	0.617
Fibrates	5.8	2.3	0.051
UAER (ug/min)	48.7(27.0–97.3)	6.4(4.7–9.7)	< 0.001

Data are presented as the median (interquartile range) for continuous variables or percentage for categorical variables

*Abbreviations: SBP* Systolic blood pressure, *DBP* diastolic blood pressure, *TC* total cholesterol, *TG* triglyceride, *HDL-C* high-density lipoprotein cholesterol, *LDL-C* Low-density lipoprotein cholesterol, *Cr* creatinine, *BMI* body mass index, *WC* waist circumference, *HbA1c* glycated hemoglobin, *FPG* fasting plasma glucose, *HOMA-IR* homeostasis model assessment of insulin resistance, *HOMA-B* homeostasis model assessment of pancreatic β-cell function, *UAER*, urinary albumin excretion rate

^a^Mann-Whitney U test or Chi-square test

### Trend analysis of clinical characteristics across HDL-C quartiles

The trend analysis of biochemical characteristics and demographics among the four subgroups of HDL-C is shown in Table 2. Patients with the highest levels of HDL-C (as reflected by the top quartile of HDL-C) had lower prevalence of microalbuminuria and lower UAER, TG levels, BMI, WC, baseline glucose levels, baseline C-peptide, Cr, HOMA-B, and HOMA-IR, but greater TC and LDL-C compared with those who had the bottom quartile of HDL-C (All *P* < 0.05). The UAER of Q4 group was obviously lower than that of Q1 to Q3 groups. The UAER from the first to fourth quartile of HDL-C was 9.4 (6.0–27.2) ug/min, 7.9 (5.3–22.3) ug/min, 9.2 (5.6–25.3) ug/min, and 6.4 (4.5–13.6) ug/min, respectively (*P* < 0.001). As expected, the prevalence of microalbuminuria in Q4 group was the lowest among the four subgroups, and the prevalence of microalbuminuria from the first to fourth quartile of HDL-C was 33.1%, 28.6%, 29.4%, and 13.5%, respectively (*P =* 0.001).

**Table 2:** Characteristics of study sample across HDL-C quartiles and trend analysis

Characteristics	Q1	Q2	Q3	Q4	*P* value
n	139	133	126	126	
Female, %	64.7	48.1	40.5	38.1	< 0.001
Age (years)	62 (56–71)	61 (55–69)	63 (53–72)	63 (54–70)	0.882
SBP (mmHg)	135 (122–150)	130 (12–145)	138 (128–151)	140 (123–155)	0.101
DBP (mmHg)	80 (75–90)	80 (75–90)	80 (78–90)	80 (75–90)	0.706
TC (mmol/L)	4.2 (3.4–4.9)	4.6 (4.0–5.5)	4.9 (4.2–5.4)	5.1 (4.3–5.9)	< 0.001
TG (mmol/L)	1.5 (1.1–2.5)	1.6 (1.1–2.5)	1.3 (1.0–1.9)	1.0 (0.7–1.4)	< 0.001
HDL-C (mmol/L)	0.85 (0.80–0.90)	1.01 (0.97–1.04)	1.17 (1.13–1.22)	1.44 (1.33–1.58)	< 0.001
LDL-C (mmol/L)	2.4 (1.9–2.9)	2.7 (2.1–3.3)	2.7 (2.3–3.3)	2.9 (2.3–3.5)	< 0.001
Cr (umol/L)	88 (79–100)	83 (75–95)	84 (74–94)	84 (74–93)	0.012
BMI (Kg/m^2^)	26.7 (24.9–29.2)	26.0 (24.3–28.6)	26.1 (24.1–28.1)	24.8 (22.6–27.3)	< 0.001
WC (cm)	99 (92–107)	97 (92–105)	95 (89–102)	94 (86–100)	< 0.001
Duration of diabetes (years)	10 (5–16)	10 (5–16)	10 (6–15)	10 (5–15)	0.525
HbA1c (%)	8.9 (7.7–10.8)	8.6 (7.2–10.2)	8.5 (7.5–10.2)	8.8 (7.6–10.1)	0.299
FPG (mmol/mol)	73.8 (48.6–86.9)	58.5 (45.4–77.0)	61.7 (50.8–77.0)	50.8 (38.8–69.4)	< 0.001
HOMA-IR	274.2 (157.3–384.9)	199.3 (128.0–341.4)	214.7 (122.4–299.0)	140.1 (80.9–232.7)	< 0.001
HOMA-B	3011.5 (2053.9–4469.8)	3086.7 (2053.4–4816.6)	2755.3 (1864.0–4361.6)	2605.2 (1639.0–4278.2)	0.012
Fasting C peptide (ng/ml)	2.1 (1.5–2.8)	1.9 (1.2–2.7)	1.8 (1.1–2.4)	1.3 (0.9–2.0)	< 0.001
Smoking (%)	31.7	24.1	20.6	19	0.012
Drinking (%)	15.1	24.1	15.9	9.5	0.333
Diabetic retinopathy (%)	31.6	37.2	33.3	30.4	0.262
Diabetes medications (%)					
Metformin	61.2	57.9	55.2	53.2	0.167
Sulfonylureas	26.6	21.8	27.2	17.5	0.182
Thiazolidinediones	12.9	15.0	12.8	12.7	0.815
Acarbose	59.7	69.9	72.0	72.2	0.027
Exenatide	1.4	2.3	1.6	0	0.281
Saxagliptin	2.9	4.5	3.2	6.3	0.246
Insulin	77.7	79.7	72.8	81.0	0.867
Antihypertensive medications (%)					
ACE inhibitor	46.8	44.4	49.6	38.1	0.291
β-Blocker	23.7	21.1	14.4	13.5	0.013
Calcium channel blockers	23.7	28.6	25.6	27.0	0.680
Lipid-lowing medications (%)					
Statins	52.5	44.4	53.6	41.3	0.207
Fibrates	3.6	4.5	2.4	2.4	0.413
UAER (ug/min)	9.4 (6.0–27.2)	7.9 (5.3–22.3)	9.2 (5.6–25.3)	6.4 (4.5–13.6)	< 0.001
Microalbuminuria (%)	33.1	28.6	29.4	13.5	0.001

Patients were divided into four groups according to the quartile (< 0.93, 0.93~ 1.08, 1.08~ 1.28, ≥ 1.28). Data are presented as the median (interquartile range) for continuous variables or percentage for categorical variables. *P* values are from nonparametric tests for trend across ordered groups, an extension of the Wilcoxon rank-sum test, and *P* < 0.05 indicates statistical significance

*Abbreviations: SBP* systolic blood pressure, *DBP* diastolic blood pressure, *TC* total cholesterol, *TG* triglyceride, *HDL-C* high-density lipoprotein cholesterol, *LDL-C* low-density lipoprotein cholesterol, *Cr* creatinine, *BMI* body mass index, *WC* waist circumference, *HbA1c* glycated hemoglobin, *FPG* fasting plasma glucose, *HOMA-IR* homeostasis model assessment of insulin resistance, *HOMA-B* homeostasis model assessment of pancreatic β-cell function, *UAER* urinary albumin excretion rate

### Logistic regression analysis

In Multivariate logistic regression models, a negative association was found between microalbuminuria and HDL-C after adjusting for SBP, DBP, age, gender, HbA1c, BMI, TC, TG, LDL-C, DR, smoking, duration of diabetes, medications, HOMA-B, and HOMA-IR (OR _Top vs. Bottom_ = 0.17, 95% CI 0.05–0.57, *P* = 0.004) (see Table 3). Medications included metformin, sulfonylureas, thiazolidinediones, acarbose, exenatide, saxagliptin, insulin, ACE inhibitors, β-Blocker, calcium channel blockers, statins, and fibrates. Besides, in the fully adjusted model 3, the results revealed that the HOMA-B was negatively associated with microalbuminuria (OR = 0.9997, 95% CI 0.9994–0.9999, *P* = 0.027) (Additional file 1: Table S1).

**Table 3:** Adjusted OR and 95% CI of microalbuminuria associated with quartiles of HDL-C

	Q2		Q3		Q4	
	OR (95% CI)	*P* value	OR (95% CI)	*P* value	OR (95% CI)	*P* value
HDL-C						
Model 1	0.85 (0.51–1.44)	0.556	0.89 (0.52–1.51)	0.654	0.36 (0.19–0.67)	0.001
Model 2	0.56 (0.21–1.48)	0.241	0.45 (0.17–1.20)	0.112	0.23 (0.08–0.71)	0.010
Model 3	0.43 (0.15–1.22)	0.111	0.34 (0.12–0.96)	0.042	0.17 (0.05–0.57)	0.004

Q2, Q3, and Q4 are compared to patients who have the bottom quartile of the HDL-C. Age and gender are adjusted in Model 1. Variables in model 1 and systolic blood pressure, diastolic blood pressure, glycated hemoglobin, total cholesterol, triglyceride, low-density lipoprotein cholesterol, diabetic retinopathy, smoking, body mass index, duration of diabetes and medications are adjusted in model 2. Variables in model 2 and homeostasis model assessment of insulin resistance, homeostasis model assessment of pancreatic β-cell function are adjusted in model 3

*Abbreviations: OR* odds ratio, *CI* confidence interval

## Discussion

In the present study, it was found that the prevalence of microalbuminuria in type 2 diabetic patients was 26.3%, which was consistent with the previous report [6]. Epidemiologic studies have shown that cardiovascular disease (CVD) is responsible for the majority deaths in T2DM, and microalbuminuria is associated with an increased CVD morbidity and mortality in patients with diabetes [18, 19]. Therefore, it is recommended that UAER should be closely monitored in diabetic patients [20].

Dyslipidemia in individuals with T2DM is very common, with a prevalence of 72–85% [21, 22]. The main diabetic dyslipidemia is low HDL-C levels due to an increased HDL catabolism rate [23], compounded by impaired HDL-C function as a result of advanced glycosylation end products [24]. The activity of lipoprotein lipase, which is an enzyme responsible for chylomicron and very low-density lipoprotein hydrolysis, is significantly reduced in patients with T2DM. In addition, plasma non-esterified fatty acid concentrations are increased due to reduced inhibition of hormone-sensitive lipase in patients with T2DM. The net result is a large pool of triacylglycerol-rich lipoproteins, which increases cholesteryl ester transfer protein (CETP) activity and facilitates triacylglycerol enrichment of HDL particles induced by CETP. As a result, hepatic lipase and HDL catabolism are promoted by the increased triacylglycerol content of HDL [23]. Our study showed that T2DM patients with microalbuminuria had lower serum HDL-C levels than those without microalbuminuria. Meanwhile, a negative relationship was found between HDL-C levels and microalbuminuria after adjusting for multiple confounding factors, including SBP, DBP, HbA1c, BMI, age, gender, TC, TG, LDL-C, DR, smoking, duration of diabetes, medications, HOMA-IR, and HOMA-B. Compared with patients who had the lowest quartile of HDL-C, there was a decreased prevalence of microalbuminuria by 83% or so among those who had the highest quartile. It was revealed that higher levels of HDL-C were associated with the decreased rates of microalbuminuria. The association between serum HDL-C levels and microalbuminuria in T2DM patients was controversial in the previous reports [12–14]. Ravid et al. reported that no correlation was found between DN progression and HDL-C levels [13]. But another report showed that higher levels of HDL-C in T2DM patients were associated with a lower risk of DN [12]. According to Tu et al.’s report, T2DM patients with healthy HDL-C levels were protected from the development of MAU [14]. These inconsistencies may be owing to differences in the patients such as ethnic backgrounds, clinical characteristics, and variation in subclass distribution of HDL-C [1, 25, 26].

The patients of our study are Chinese, an ethnic population more prone to low HDL-C levels and microalbuminuria than their Caucasian counterparts. Wheat and rice, staple foods of the Chinese Han population, and high-carbohydrate diets have been associated with the decreased HDL-C concentration [26, 27]. Some studies found no association between serum HDL-C levels and microalbuminuria, which may be due to higher overall serum HDL-C levels of patients compared with Chinese subjects. Another explanation for discrepancies regarding HDL-C and microalbuminuria associations lies in HDL-C subclassification. HDL-C is subclassified into two major fractions based on density: HDL2 and HDL3 [28]. Vasoprotective effects are predominantly attributed to HDL2, while HDL3 is implicated in atherosclerosis and could predict microalbuminuria incidence [1, 25]. Nuclear magnetic resonance analysis revealed that patients with DN were usually characterized by decreased HDL2 and increased HDL3, suggesting that low levels of the HDL2 subtype accounted for majority of the HDL-C loss in DN. Therefore, the lack of associations between HDL-C levels and microalbuminuria in other studies may be possibly due to differential changes in HDL2 and HDL3 subtypes in opposite directions on exacerbating nephropathy [25]. Our study found that serum HDL-C levels in patients with T2DM were negatively correlated with microalbuminuria. This may be explained that the decrease of HDL-C level in our study patients is mainly caused by the significant decrease of HDL2 subtypes, resulting in a lack of renal protection of HDL2.

Although the precise mechanism for the inverse relationship is unknown, there are several speculative mechanisms to interpret how HDL-C influences the development of microalbuminuria. Firstly, the reversal cholesterol transport process may be hindered by HDL-C functional impairment or insufficiency, and glomerular sclerosis and renal tubular interstitial injury are induced [29]. Secondly, reduced HDL-C antioxidative ability results in increased systemic oxidative stress and circulatory oxidized LDL, an initiator of immune-mediated diabetic nephropathy [30]. Finally, lower HDL-C levels might decrease glucose uptake in skeletal muscle and induce pancreatic β-cell dysfunction. Beta cell dysfunction leads to hyperglycemia and metabolic disorder which damage endothelial and mesangial cells in the glomerulus [11, 31].

In addition, our study showed that patients with type 2 diabetes who developed microalbuminuria were more likely to have higher SBP, DBP, TG, BMI, HbA1c, HOMA-IR and prevalence of DR, but lower HDL-C and HOMA-B than those who did not. Multiple logistic regression analysis revealed that only HDL-C and HOMA-B were significantly associated with microalbuminuria. Poor pancreatic β-cell function has also been reported to be associated with microalbuminuria in previous studies [18, 32]. It is widely believed that the pathogenic relationship of β-cell dysfunction and microalbuminuria is associated with hyperglycemia, since the endothelial cells and mesangial cells are damaged in the glomerulus [18]. Poor glycemic control, hypertension, lipid abnormalities, obesity, insulin resistance, presence of DR, smoking, male sex, increasing age, and long duration of diabetes are risky factors for diabetic nephropahty [1]. In the present study, however, we found no association of HbA1c levels, blood pressure, serum TC, TG, LDL-C levels, or other above factors with microalbuminuria. The different results may be attributed to alternative sample size, ethnic groups, or clinical characteristics. The sample size in our study was not large, which might increase the chance of type 2 error and result in a false negative. Besides, we presumed that blood glucose, blood pressure, TC, TG, and LDL-C might not have been statistically significant in our study because those were well controlled by medications.

To our knowledge, there have been few studies adjusting for the confounding factors of fasting C-peptide-derived data of islet β-cell function and insulin resistance to evaluate the association between HDL-C and microvascular complications in T2DM patients. C-peptide is a by-product of insulin synthesis and released at equal levels. Therefore, to avoid inaccuracy of HOMA-IR and HOMA-B caused by the prescribed insulin and other treatments in T2DM patients, fasting plasma C-peptide was used instead of fasting insulin [17]. Previous researches have showed that insulin resistance and islet β-cell function are also implicated in the pathogenesis of DN in addition to poor glycemic control [18, 33]. Therefore, adjustment for these key confounding factors allows for accurate estimation of the independent effect of serum HDL-C levels on microalbuminuria in T2DM.

Nevertheless, our study also has several limitations. Firstly, the sample size was a bit small. Secondly, it is possible that subtypes of HDL-C have different relationships with diabetic microvascular outcomes. Due to limited conditions in this study, it was unable to measure the subtypes of HDL-C such as HDL2 and HDL3. Thirdly, the cross-sectional nature of this study limited the ability to infer causation or directionality of relationships. Finally, although logistic regression was utilized and multivariable-adjusted relationships were reported, it remained difficult to adjust all potential confounding factors including serum uric acid and macrovascular complications. Hence, further prospective and interventional studies that cover all potential confounding factors and HDL-C subtype testing are required, so as to provide a more accurate assessment of the relationship of serum HDL-C levels with microalbuminuria in T2DM.

## Conclusion

Our study may have potential clinical implications. The findings provided evidence that HDL-C was an independent factor related to DN. And the rusults strengthened the hypothesis that T2DM patients with a higher HDL-C levels might be protected against DN. Thus, lower serum HDL-C levels in T2DM patients would remind clinicians of possible microalbuminuria and DN, and appropriate management may be taken to improve HDL-C levels.

## Additional file

Additional file 1: **Table S1.** Association of HDL-C levels with microalbuminuria after adjusting for clinical variables (model 3). (DOCX 44 kb)

## Abbreviations

BMI: Body mass index
CI: Confidence interval
Cr: Creatinine
DBP: Diastolic blood pressure
DN: Diabetic nephropathy
DR: Diabetic retinopathy
FPG: Fasting plasma glucose
HbA1c: Glycated hemoglobin
HDL-C: High-density lipoprotein cholesterol
HOMA-B: Homeostasis model assessment of pancreatic β-cell function
HOMA-IR: Homeostasis model assessment of insulin resistance
LDL-C: Low-density lipoprotein cholesterol
OR: Odds ratio
SBP: Systolic blood pressure
T2DM: Type 2 diabetes mellitus
TC: Total cholesterol
TG: Triglyceride
UAER: Urinary albumin excretion rate
WC: Waist circumference
