# Supplementary material for: Association of serum high-density lipoprotein cholesterol with microalbuminuria in type 2 diabetes patients
Source: Lipids Health Dis. 2018 Oct 5;17:229. doi: 10.1186/s12944-018-0878-2 (PMC6173850; doi:10.1186/s12944-018-0878-2)
Supplement: Supplementary file 1 — Table S1. Association of HDL-C levels with microalbuminuria after adjusting for clinical variables (model 3). (DOCX 44 kb) [file 12944_2018_878_MOESM1_ESM.docx]

**Table S1.** Association of HDL-C levels with microalbuminuria after adjusting for clinical variables (model 3)

| Variables | Coefficient | OR /EXP (coefficient) | 95% CI | *P* value |
| --- | --- | --- | --- | --- |
| HDL-C (Q1, reference) |  |  |  |  |
| HDL-C (Q2) | -0.840 | 0.43 | 0.15-1.22 | 0.111 |
| HDL-C (Q3) | -1.070 | 0.34 | 0.12-0.96 | 0.042 |
| HDL-C (Q4) | -1.782 | 0.17 | 0.05-0.57 | 0.004 |
| Age | 0.007 | 1.01 | 0.97-1.05 | 0.737 |
| Gender | 0.087 | 1.09 | 0.41-2.88 | 0.860 |
| SBP | 0.016 | 1.02 | 0.99-1.05 | 0.288 |
| DBP | -0.015 | 0.99 | 0.94-1.04 | 0.581 |
| HbA1c | 0.003 | 1.00 | 0.81-1.25 | 0.976 |
| TC | -0.401 | 0.67 | 0.33-1.36 | 0.266 |
| TG | 0.285 | 1.33 | 0.94-1.88 | 0.106 |
| LDL-C | 0.598 | 1.82 | 0.71-4.66 | 0.213 |
| DR | 0.256 | 1.29 | 0.76-2.20 | 0.344 |
| Smoking | 0.016 | 1.02 | 0.37-2.79 | 0.975 |
| BMI | -0.071 | 0.93 | 0.82-1.05 | 0.260 |
| Duration of diabetes | 0.005 | 1.01 | 0.95-1.06 | 0.855 |
| Metformin  Sulfonylureas  Thiazolidinediones  Acarbose  Exenatide  Saxagliptin  Insulin | 0.555  -0.168  0.031  -0.419  0.041  0.022  -0.004 | 1.74  0.85  1.03  0.66  1.05  1.01  1.00 | 0.77-3.95  0.32-2.21  0.30-3.52  0.30-1.44  0.31-3.68  0.28-3.45  0.33-3.05 | 0.183  0.732  0.961  0.296  0.972  0.991  0.995 |
| ACE inhibitor  β-Blocker  Calcium channel blockers | 0.814  -0.972  -0.497 | 2.26  0.38  0.61 | 0.94-5.45  0.12-1.20  0.24-1.57 | 0.070  0.099  0.304 |
| Statins  Fibrates | 0.075  1.327 | 1.08  3.77 | 0.49-2.38  0.52-27.30 | 0.854  0.189 |
| HOMA-IR | 0.001 | 1.001 | 0.999-1.003 | 0.558 |
| HOMA-B | -0.0003 | 0.9997 | 0.9994-0.9999 | 0.027 |

The multivariate model (model 3) is adjusted for age, gender, SBP, DBP, BMI, HbA1c, TC, TG, LDL-C, DR, smoking, duration of diabetes, medications, HOMA-B, and HOMA-IR. Abbreviations: OR, odds ratio; CI, confidence interval; HDL-C, high-density lipoprotein cholesterol; SBP, systolic blood pressure; DBP, diastolic blood pressure; HbA1c, glycated hemoglobin; TC, total cholesterol; TG, triglyceride; LDL-C, low-density lipoprotein cholesterol; DR, diabetic retinopathy; BMI, body mass index; HOMA-IR, homeostasis model assessment of insulin resistance; HOMA-B, homeostasis model assessment of pancreatic β-cell function;
